# Draft Genome Sequence of Thiostrepton-Producing *Streptomyces azureus* ATCC 14921

**DOI:** 10.1128/genomeA.01183-15

**Published:** 2015-10-22

**Authors:** Kengo Sakihara, Jumpei Maeda, Kosuke Tashiro, Yasuhiro Fujino, Satoru Kuhara, Toshihisa Ohshima, Seiya Ogata, Katsumi Doi

**Affiliations:** a Microbial Genetic Division, Institute of Genetic Resources, Faculty of Agriculture, Kyushu University, Fukuoka, Japan; b Department of Bioscience and Biotechnology, Faculty of Agriculture, Kyushu University, Fukuoka, Japan; c Division for Arts and Science, Faculty of Arts and Science, Kyushu University, Fukuoka, Japan; d Department of Biomedical Engineering, Faculty of Engineering, Osaka Institute of Technology, Osaka, Japan

## Abstract

*Streptomyces azureus* ATCC 14921 belongs to the *Streptomyces cyaneus* cluster and is known to be a thiostrepton producer. Here, we report a draft genome sequence for this strain, consisting of 350 contigs containing a total of 8,790,525 bp, 8,164 predicted coding sequences, and a G+C content of 70.9%.

## GENOME ANNOUNCEMENT

*Streptomyces* species are Gram-positive aerobic mycelial bacteria belonging to the phylum *Actinobacteria*. They are useful for their capacity to produce large numbers of secondary metabolites and are also interesting subjects for the study of morphological differentiation (1). Pock formation involving conjugative plasmids is a typical physiological characteristic of the morphological differentiation of *Streptomyces* species (2, 3).

*Streptomyces azureus* ATCC 14921 is categorized in the blue-spore *Streptomyces cyaneus* cluster and is known to be a thiostrepton producer (4). The thiostrepton resistance gene (*tsr*), which is a selective marker often used in the genetic engineering of actinomycetes, was isolated from the strain (5, 6). *S. azureus* contains the pock-forming conjugative plasmid pSA1.1 (7), and spontaneous development of pocks in the strain is caused by the action of the plasmid and lysogenic phage SAt2 (8). The genome sequence of *S. azureus* may shed light on the mechanism of pock formation and may also be useful in comparative studies of morphological and metabolic differentiation among blue-spore *Streptomyces* species.

A sample was prepared for sequencing by growing *S. azureus* ATCC 14921 aerobically overnight at 28°C in tryptic soy broth (TSB) (Oxoid). The genomic DNA was then extracted and purified as we described previously (9). The prepared genome was sequenced using MiSeq (Illumina) and Ion PGM (Thermo) and assembled using the Microbial Genome Annotation Pipeline (MiGAP) (http://www.migap.org/) (10).

The genomic DNA included a total of 8,790,525 bp and was sequenced using the whole-genome shotgun strategy, which generated 3,669,353 reads and achieved approximately 62-fold coverage. Assembly of all the reads resulted in 156 contigs (>100 bp), with an *N*_50_ contig size of 85,729 bp. Genome annotation of the obtained scaffolds was performed using MetaGeneAnnotator version 1.0 and NCBI BLAST version 2.2.18 against a nonredundant protein sequence database. The genome of *S. azureus* ATCC 14921 has a G+C content of 70.9%, and annotation using the COG, RefSeq, and TrEMBL databases with tRNAscan-SE version 1.23 (11) and additional manual inspection revealed 8,164 predicted coding regions, 68 tRNA genes, and 3 rRNA genes.

The gene cluster for thiostrepton biosynthesis was annotated within contig SAZU364 and showed strong similarity to the thiostrepton biosynthesis cluster of *Streptomyces laurentii* ATCC 31255 (12). The *tsr* gene is located between the ABC transporter genes and two-component response system genes within contig SAZU036. Plasmid pSA1.1 was integrated adjacent to the tRNA^Ile^ gene in contig SAZU077. We suggest pock formation is caused by competitive inhibition between products of the *spi* gene in pSA1.1 and the *spoIIIE*- or *ftsK*- like gene products (13). However, no genes homologous to *spoIIIE*- or *ftsK* have been annotated in the genome. An alternative mechanism for pock formation in *S. azureus* should therefore be considered. Within contig SAZU025, a gene cluster encoding phage-related proteins, including the tail protein, capsid and scaffold proteins, terminase, and integrase, was annotated. It appears these genes encoding defective phage SAt2 might produce a lytic zone in spontaneously developing pocks.

### Nucleotide sequence accession number.

The *S. azureus* ATCC 14921 genome sequence and annotation data have been deposited in the DDBJ/EMBL/GenBank under the accession no. BBYS00000000.

## References

[1] Chandra G, Chater KF 2014 Developmental biology of *Streptomyces* from the perspective of 100 actinobacterial genome sequences. FEMS Microbiol Rev 38:345–379. doi:10.1111/1574-6976.12047. 24164321 PMC4255298

[2] Thoma L, Vollmer B, Muth G 2015 Fluorescence microscopy of *Streptomyces* conjugation suggests DNA-transfer at the lateral walls and reveals the spreading of the plasmid in the recipient mycelium. Environ Microbiol, in press. doi:10.1111/1462-2920.13027. 26286483

[3] Doi K, Ohyama Y, Yokoyama E, Nishiyama T, Fujino Y, Nagayoshi Y, Ohshima T, Ogata S 2012 Expression analysis of the *spi* gene in the pock-forming plasmid pSA1.1 from *Streptomyces azureus* and localization of its product during differentiation. Appl Microbiol Biotechnol 95:707–716. doi:10.1007/s00253-012-4000-9. 22526776

[4] Trejo WH, Bennett RE 1963 *Streptomyces* species comprising the blue-spore series. J Bacteriol 85:683–684. 10.1128/jb.85.3.676-690.1963 PMC278201 14042949

[5] Janssen GR, Bibb MJ 1990 Tandem promoters, *tsr*p1 and *tsr*p2, direct transcription of the thiostrepton resistance gene (*tsr*) of *Streptomyces azureus*: transcriptional initiation from *tsr*p2 occurs after deletion of the -35 region. Mol Gen Genet 221:339–346. 2381416 10.1007/BF00259397

[6] Wada K, Kobayashi J, Furukawa M, Doi K, Ohshiro T, Suzuki H 3 9 2015 A thiostrepton resistance gene and its mutants serve as selectable markers in *Geobacillus kaustophilus* HTA426. Biosci Biotechnol Biochem, in press. doi:10.1080/09168451.2015.1079478. 26333661

[7] Yokoyama E, Matsuzaki Y, Doi K, Ogata S 1998 Gene encoding a replication initiator protein and replication origin of conjugative plasmid pSA1.1 of *Streptomyces cyaneus* ATCC 14921. FEMS Microbiol Lett 169:103–109. doi:10.1111/j.1574-6968.1998.tb13305.x. 9851040

[8] Yamada S, Suenaga H, Doi K, Yoshino S, Ogata S 2003 Effects of UV dose on formation of spontaneously developing pocks in *Streptomyces azureus* ATCC 14921. Biosci Biotechnol Biochem 67:797–802. doi:10.1271/bbb.67.797. 12784620

[9] Doi K, Saeki MC, Ono Y, Ogata S 1995 Plasmid formation and its relation to the formation of spontaneously developing pocks in *Streptomyces azureus* ATCC 14921. J Appl Microbiol 79:237–251.

[10] Noguchi H, Taniguchi T, Itoh T 2008 MetaGeneAnnotator: detecting species-specific patterns of ribosomal binding site for precise gene prediction in anonymous prokaryotic and phage genomes. DNA Res 15:387–396. doi:10.1093/dnares/dsn027. 18940874 PMC2608843

[11] Schattner P, Brooks AN, Lowe TM 2005 The tRNAscan-SE, snoscan and snoGPS Web servers for the detection of tRNAs and snoRNAs. Nucleic Acids Res 33:W686–W689. doi:10.1093/nar/gki366. 15980563 PMC1160127

[12] Kelly WL, Pan L, Li C 2009 Thiostrepton biosynthesis: prototype for a new family of bacteriocins. J Am Chem Soc 131:4327–4334. doi:10.1021/ja807890a. 19265401

[13] Doi K, Ono Y, Yokoyama E, Tsukagoe Y, Ogata S 1998 Whole sequence of *spoIIIE*-like, sporulation-inhibitory, and transfer gene (*spi*) in a conjugative plasmid, pSA1.1, of *Streptomyces azureus* and detection of *spi*-like gene in the actinomycete chromosome. Biosci Biotechnol Biochem 62:1597–1600. doi:10.1271/bbb.62.1597. 9757567

